# Special Hospitals: III. Children's

**Published:** 1893-09-09

**Authors:** 


### The Institutional Workshop.

### HOSPITAL ADMINISTRATION.

### III.—SPECIAL HOSPITALS: CHILDREN'S.

It will be seen from the table which follows how large a portion of hospital care is now extended to children in special hospitals. There are twelve children's hospitals in our list, and it will be noticed that the work done by some of them is as great, and entails nearly as large an outlay, as that of a general hospital. The number of beds these hospitals contain varies from 182 at the Hospital for Sick Children, Great Ormond Street, to 24 at the Belgrave Hospital. We have included three institutions which are practically homes for incurable or chronic children, because the work which these latter institutions do is most helpful to the hospitals, as well as beneficial to the children and their parents. Another feature to be noticed is that the number of out-patients admitted to treatment seems to bear no relation to the number of beds, if we may judge from the fact that the Belgrave Hospital, with 24 beds, treated 2,611 out-patients, whilst at Paddington Green Hospital, which contains 29 beds, no fewer than 10,837 were treated in the out-patient department in 1892. There is a marked difference between the cost per bed at the Belgrave Hospital and at the Paddington Green Hospital for Children. The question of cost is a most important one, but space will not permit us to deal with it here, although the foregoing facts illustrate the difficulties which inquirers have to face when they attempt to judge impartially between the relative economy and efficiency of these institutions by the figures supplied by the hospital authorities.

We laid it down last year, and see no reason to change our opinion, that 15 per cent. should be the maximum percentage of cost of administration to that of maintenance at a children's hospital. This is so because there are no more popular institutions than hospitals for children, and none, probably, which elicit greater sympathy or a larger proportion of spontaneous support. Hence it cannot fairly be maintained that it is more difficult to raise money for the children's hospitals than for the general hospitals, and so 15 per cent. is undoubtedly the maximum expenditure upon management which should be permitted by those who are responsible for the management of a children's hospital. This statement must be qualified to the extent of permitting an additional 5 per cent. to be expended upon management in the case of those children's hospitals which are situated in the heart of poor neighbourhoods, far away from the public gaze which receive very little spontaneous assistance in consequence. Of all the hospitals those for children should certainly be situated near the homes of the patients, who necessarily come in greatest numbers from the poorest districts. Hence a wise philanthropist will encourage the managers of a children's hospital so situated, should it be found necessary to expend 20 per cent. upon management for the causes here set forth.

The East London Hospital for Children spends more than any other upon management. Thus the percentage of the cost of administration to that of maintenance during 1892 was 18·876, and the percentage of cost of administration to total expenditure was 15·879, the former figures representing an increase of ½ per cent. in the expenditure as compared with the year 1891. The North-Eastern Hospital, on the other hand, though situated in a very poor district, expended about 2 per cent. less, the percentage of cost of administration to that of maintenance being 16·190, and the percentage of cost of administration to total expenditure being 13·934. These figures are highly creditable to the authorities of the latter institution, which is very seriously hampered for want of funds to erect necessary buildings to enable the committee to provide necessary accommodation for the ever-increasing number of children who apply for admission. These additional funds should be given without further delay, and we hope that before next year comes round sufficient money will be forthcoming to enable the new wing to be built and occupied.

The Paddington Green Hospital, which has been closed for a number of months during the present year, has reduced the percentage of cost of administration from 20 per cent. in 1891 to 16·852 per cent. in 1892, the percentage of administration being 14·422. This is a considerable improvement, but a still further reduction must be made if this hospital is to take a good place for sound management, seeing the comparative ease with which West-end charities like this raise their money. It is no small feather in the cap of the North-Eastern management that they should expend nearly ¾ per cent. less upon the cost of administration to maintenance than the Paddington Green Hospital.

Metropolitan Children's Hospitals.

| Name of Hospital. | Beds for Occupation. | Average Number Occupied. | Total In-patients Admitted. | Total Out-patients Admitted. | Percentage of cost of Administration to that of Maintenance. | Percentage of Administration Total to Expenditure. |
|---|---|---|---|---|---|---|
| Hospital for Sick Children, Great Ormond St. | 182 | 120 | 1,518 | 21,045 | 14·349 | 12·550 |
| Victoria Hospital for Children... ... ... | 116 | 95 | 1,375 | 17,075 | 15·545 | 13·454 |
| East London Hospital ... ... ... ... | 102 | 77 | 1,257 | 7,296 | 18·876 | 15·879 |
| Alexandra Hospital for Hip Disease ... ... | 81 | 79 | 147 | 90 | 10·522 | 9·520 |
| Evelina ... ... ... ... ... ... ... | 66 | 51 | 577 | 5,201 | 5·851 | 5·528 |
| North-Eastern ... ... ... ... ... ... | 55 | 41 | 556 | 14,233 | 16·190 | 13·934 |
| Cheyne for Incurables ... ... ... ... | 50 | 50 | 72 | none | 8·394 | 7·716 |
| Home for Sick Children, Sydenham ... ... | 45 | 44 | 218 | 1,395 | 2·682 | 2·612 |
| Home for Incurable Children, Maida Vale ... | 30 | 29 | 33 | none | 7·528 | 6·953 |
| Paddington Green ... ... ... ... ... | 29 | 9 | 137 | 10,837 | 16·852 | 14·422 |
| Belgrave ... ... ... ... ... ... ... | 24 | 21 | 196 | 2,611 | 6·075 | 5·727 |
| The Vine, Sevenoaks ... ... ... ... | 24 | 18 | 37 | none | 3·692 | 3·662 |

All the other hospitals are within the 15 per cent. limit, the Evelina only expending so little as 5·851 per cent. on administration to maintenance, a fact due in no small degree, we believe, to the interest taken in this institution by the Rothschild family. It would not be a bad idea for other families of influence and importance to take a children's hospital under their wing, and so to enable its managers to do the work much more economically, to a much fuller extent than is now possible.

It may have been observed, as we pointed out last year, that in the case of a consumption hospital the cost of administration doubled as the beds diminished, but in the case of a children's hospital it would appear, from the above table, that the fewer the number of beds the smaller is the cost of administration, excluding, however, the Paddington Green Hospital, which is too high, and the Evelina, which is remarkably low. Only 3·692 per cent. is spent on management at the Vine, Sevenoaks, and at the Home for Sick Children, Sydenham, the percentage is even less, being 2·682 per cent. In the two last institutions the percentage of cost of administration to that of *(a)* maintenance and *(b)* total expenditure is almost identical. Is it possible to hope that, as time goes by and more attention is directed to management, other and larger institutions may find it possible to approximate the percentage in the same way? At present, as we pointed out in a former article, there is a difference of, on an average, quite 2 per cent. between these percentages at nearly all hospitals, no matter to what class they may belong. Possibly the smaller institutions are to a greater extent under voluntary management, where salaries are reduced to a minimum, and personal service is an asset which forms a large item of value in the course of the year, though it is not brought to account in the balance-sheet.

